# A bibliometric analysis of global research output on health and human rights (1900–2017)

**DOI:** 10.1186/s41256-018-0085-8

**Published:** 2018-10-22

**Authors:** Waleed M. Sweileh

**Affiliations:** Department of Physiology, Pharmacology/Toxicology, Division of Biomedical Sciences, College of Medicine and Health Sciences, An-Najah National University, Nablus, Palestine

**Keywords:** Health, Human rights, Bibliometric analysis

## Abstract

**Background:**

Baseline data on global research activity on health and human rights (HHR) needs to be assessed and analyzed to identify research gaps and to prioritize funding and research agendas. Therefore, the aim of this study was to assess the growth of publications and research pattern on HHR.

**Methods:**

A bibliometric methodology was used. Literature on HHR was retrieved using SciVerse Scopus for the study period from 1900 to 2017. Nine different search scenarios with different keyword combinations were used to retrieve the required documents. All types of documents published in peer-reviewed journals, including editorials, were included. The search strategy was validated.

**Results:**

In total 6513 documents were retrieved with an *h*-index of 88 and an average of 9.8 citations per document. Publications on HHR field started as early as 1950 but showed a steep rise in the past two decades. Visualization of author keywords revealed that HIV/ AIDS, mental health, maternal and reproductive health, violence, ethics, torture, and refugees were most commonly encountered keywords. The journal *“Health and Human Rights”* was most active (*n* = 467; 7.2%) in this field. However, documents that appeared in *The Lancet* received the highest impact (29.5 citations per document). The United States of America produced the most in this field (*n* = 1817; 27.9%). Researchers in the region of Americas participated in approximately 45% of the retrieved documents while researchers in the Eastern Mediterranean region had the least contribution (2.5%). Researchers in high-income countries contributed to approximately 78% of the retrieved documents while researchers in low-income countries contributed to less than 5% of the retrieved documents. When data were standardized by population size, the research output from high-income countries was approximately four documents per one million inhabitants. For middle-income countries, the research output was 0.3 document per one million inhabitants. For low-income countries, the research output was 0.5 document per one million inhabitants.

**Conclusions:**

Differential research productivity on HHR was seen among scholars in different world regions. World countries need to encourage and strengthen research on HHR in order to achieve the goals set in international agreements of human rights.

**Electronic supplementary material:**

The online version of this article (10.1186/s41256-018-0085-8) contains supplementary material, which is available to authorized users.

## Background

The 1946 World Health Organization (WHO) Constitution defined health as “a state of complete physical, mental and social well-being and not merely the absence of disease or infirmity” [1]. International agreements such as *International Covenant on Economic, Social and Cultural Rights* (Article 12) defined the right to health as “the right of everyone to the enjoyment of the highest attainable standard of physical and mental health” [2]. International agreements and declarations urge all nations to promote different aspects of human rights and emphasized the interrelationship between health and human rights (HHR) [3]. The struggle for human rights was endorsed by several social movements including ones pertaining to women’s rights [4–6].

The continuous work and involvement of United Nations (UN) in global health led to the emergence of the new global agenda for the period 2015–2030 to replace the Millennium Development Goals (MDGs). In September 2015, the UN adopted the 2030 Sustainable Development Goals (SDGs) agenda, which included 17 goals measured through 169 targets [1]. The 2030 agenda is in accordance with the UN charter and with the Universal Declaration of Human Rights. The SDGs focus on various subjects such as ending poverty and hunger, reducing inequalities, improving education, and gender equality. The concept of HHR is fundamental in 2030 SDG agendas despite that it was not phrased in a direct way [7–9]. The third goal in SDGs deals directly and comprehensively with health and was operationalized through nine targets. The SDGs aim to achieve human rights for all regardless of “race, color, sex, language, religion, political or other opinion, national or social origin, property, birth, disability or other status”.

Achieving the 2030 agenda requires active research to identify potential research gaps and research domains mostly discussed by researchers in each country. Actually, research on HHR might affect global foreign policy agendas and action plans set by international groups that might pressure for HHR in certain world regions. Ignoring research on HHR might increase vulnerability, amplifies discrimination, and health inequalities. On the contrary, research on HHR can shed light, and consequently, reduce health and social costs, improve social cohesion, which will protect public health and human rights.

At the academic level, many graduate programs and academic departments specialized in HHR have been created. The overall mission of researchers and academic specialists in the field of HHR is to endorse the concept of HHR through international conferences, peer–reviewed publications, launching scientific journals dedicated for HHR, and training health workers in the field of HHR. In many universities, these tasks are usually carried out by public health experts and by those in the field of law. Academics and researchers need to participate actively in achieving the general principles of health for all by pointing gaps in the field of HHR and prioritizing action plans to promote positive change in healthcare, particularly in low and middle-income countries (LMIC). Researchers can also advocate HHR for minorities and for neglected and marginalized people by creating a public debate that can ultimately influence political campaigns and political agenda toward universal health coverage [10]. The importance of research and advocacy of the HHR was made clear by launching several journals in the field of HHR. The size, growth pattern, and mapping of global research productivity on HHR had not been addressed before despite that systematic reviews, editorials, and research articles in this field had been published [11–13]. Furthermore, assessment of global research output regarding several health aspects in vulnerable groups of people has been published but not from a human right perspective [14–17]. Therefore, the objective of this study was to assess the volume, growth, and research trends on HHR.

## Methods

### Database used

In the current study, publications on HHR were retrieved using the well-known database, SciVerse Scopus which has several advantages over other known databases [18]. Scopus is commonly used as a reference database for bibliometric analysis [19–21] which is defined as the use of several mathematical and statistical techniques in order to assess the volume, scientific impact, growth, and research trends in a particular topic [22]. Scopus has several functions that facilitate bibliometric analysis. For example, Scopus sorts publications based on the number of citations or date or country or author or journal or institution. Furthermore, Scopus counts the number of citations for any set of documents and calculates the Hirsh-index (*h*-index) which is used as a measure of the scientific impact for any set of documents [23].

### Search strategy and keywords

The strategy used to retrieve relevant publications consisted of 12 steps (Additional file 1). The first nine steps consisted of different search queries that were ultimately combined to eliminate duplicate documents. Keywords used in the search strategy included phrases such as “health right(s)” or “right(s) to health” in title or abstract. Furthermore, documents published in journals specialized in HHR were also retrieved since documents in these journals are supposed to be relevant to the theme of the study.

### Exclusion of specific keywords

In Scopus, both quotation marks and asterisks are used to enhance accuracy and comprehensiveness of the results. Furthermore, Scopus allows researchers to limit the search by name of journal or affiliation of the author. All these functions, once used, can add up to the accuracy of the search strategy. Despite that, the possibility of false positive results is always present. Therefore, an exclusion step was added to search strategy to eliminate false positive documents. The exclusion step included keywords such as human right ventricle/atrium Therefore, a set of keywords were used in the exclusion step to exclude documents containing such terms.

### Exclusion based on year or type of document

In this study, documents published in 2018 were excluded and therefore the study included all times from 1900 to December 2017. Furthermore, only documents published in peer-reviewed journals were included. Therefore, research articles, review articles, editorials, letters, notes, short surveys, and conference papers were included in the analysis while books and book chapters were excluded. No language restrictions were made on the retrieved articles.

### The validity of search strategy

The validation method used in this study was based on comparing the number of documents obtained through the current search strategy with the number of documents obtained from the personal Scopus profile for the top 10 active authors. The validation process adopted in the current study was fully explained in previously published article authored by the same research group [24]. The comparison was made through interclass correlation test using reliability testing in Statistical Package for Social Sciences 21. Interclass correlation coefficient obtained was > 95% with a *p* < 0.01 indicative of the high validity of the search strategy. The second test for validity of the search strategy was the manually review of the top 100 cited documents of the retrieved articles to ensure the absence of false positive results. The manual review of the top 100 cited articles showed no false positive results indicative of the high validity of the implemented search strategy. A third method to test for validity was to manually check the most active journals to make sure that none was in a field outside the scope of HHR. All methods that we applied to test for validity showed high validity indicative of the minimum degree of false positive results and the high level of accuracy (absence of false positive or false negative results) in the number of the retrieved articles.

### Bibliometric indicators

Retrieved documents were analyzed and the following bibliometric indicators were obtained: top 10 active countries, institutions, and journals. Scopus looks into the affiliation of countries and institutions in the retrieved documents and counts the number of documents in which the affiliation of a certain country or institution was present. Therefore, listing the top 10 active countries was based on the presence of country name in the affiliation of the authors regardless of the role of the author affiliated with that country. The research output from each country represents the sum of documents published by international research collaboration (inter-country collaboration) and documents published by intra-country research collaboration.

This, of course, creates an overlap when listing top 10 active countries because one document might have different authors with different country and institutional affiliations.

### Visualization and mapping

Author keywords were analyzed and visualized using VOSviewer software [25]. The geographical distribution of publications was mapped using ArcGIS 10.1 software [26]. The analysis also included distribution of publications based on WHO classification of world regions [27] and World Bank country classification based on 2016 national income [28]. Graphics of bar charts were made using Statistical Package for Social Sciences software (SPSS version 21 for windows). All data presented in this study were obtained by analysis of data retrieved on August 28, 2018.

## Results

### Types of the retrieved documents

In total, 6513 journal documents were retrieved. The bulk of retrieved documents were research articles (*n* = 4527; 69.5%). Other types of retrieved documents include review articles (*n* = 974; 15.0%), editorials (*n* = 321; 4.9%), research notes (*n* = 246; 3.8%), conference papers (*n* = 168; 2.6%), letters (*n* = 152; 2.3%), short surveys (*n* = 111; 1.7%) and articles in press (*n* = 14; 0.2%).

### Annual growth of publications

**Fig. 1:**
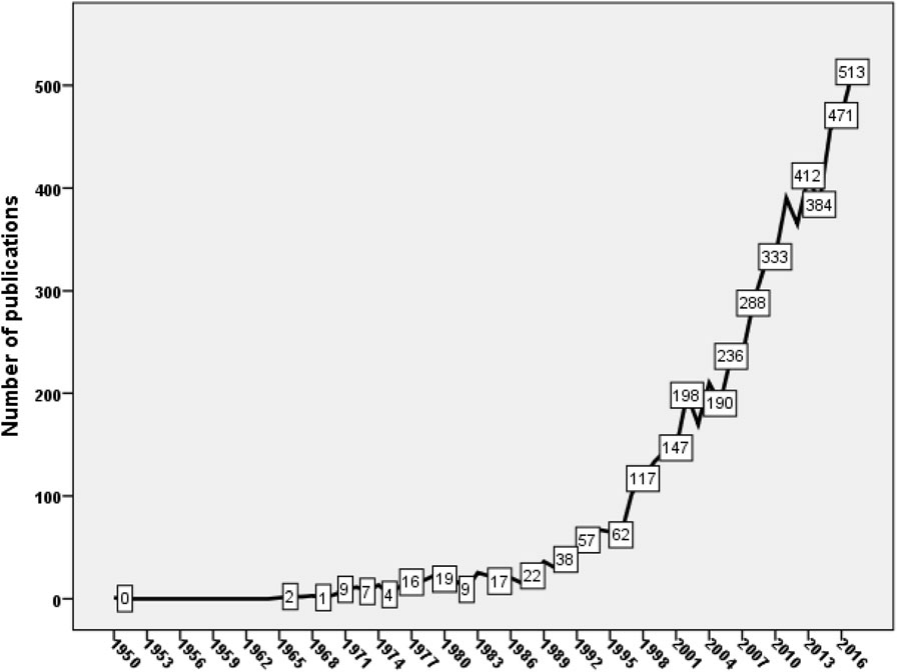
Annual growth of publications on HHR (1900–2017)

The oldest document on HHR was published in 1950 in *Le Médecin généraliste de France* [29]. The number of publications remained close to zero between 1951 and 1965 (Fig. 1). A very small increase in the number of publications was seen between 1966 and 1996. A steep noticeable rise in the number of publications was observed after 1997. The maximum number of publications was recorded in 2017 with 513 publications. The retrieved documents had an average *h*-index of 88. The retrieved documents received 64,002 citations, a mean of 9.8 citations per document.

### Visualization of author keywords

**Fig. 2:**
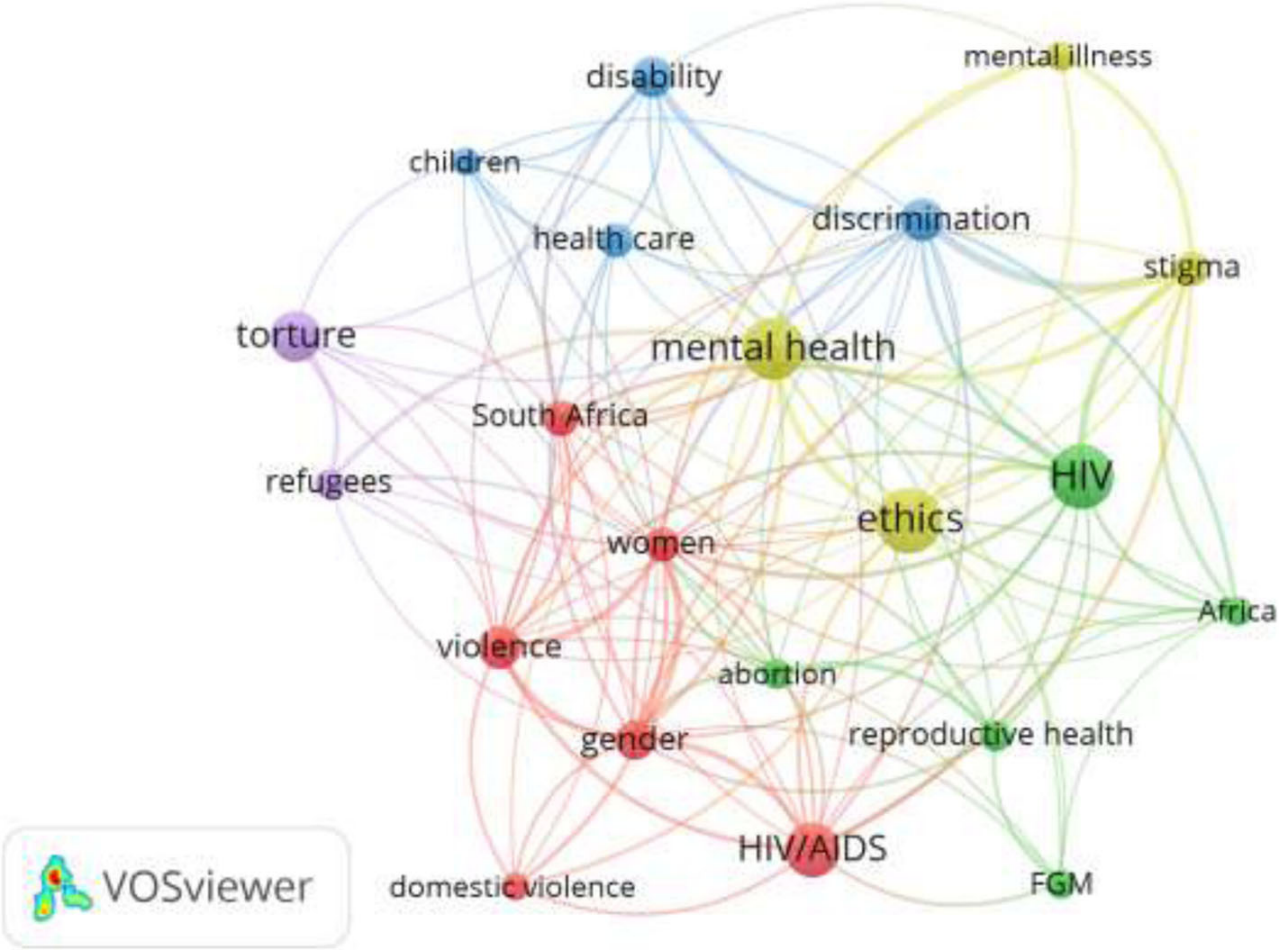
Network visualization map of keywords with minimum occurrences of 30 times. The size of the circle is a relative representation of number of occurrences with larger size indicating higher frequency of occurrences

Visualization of author keywords with a minimum occurrence of 30 times revealed that keywords such as HIV/ AIDS, mental health/illness, women/maternal health, sexual health, female genital mutilation, violence, ethics, torture, Africa, and refugees were most commonly encountered author keywords (Fig. 2). When these terms were further examined in the title and/or abstract of retrieved documents, the number of publications obtained were 912 (14.0%) documents for HIV/AIDS, 810 (12.4%) for sexual and reproductive health, and 730 (11.2%) for mental health.

### Top 10 active journals

The top 10 active journals in publishing documents on HHR were shown in Table 1. The most active journal was *“Health and Human Rights”* with 467 (7.2%) documents followed by *“BMC International Journal of Health and Human Rights”* (*n* = 388; 6.0%). However, documents published in *The Lancet* received the highest number of citations (29.5 citations per document).

**Table 1:** Top 10 active journals in publishing documents on HHR

Rank	Journal	Frequency	% (*N* = 6513)
1st	*Health And Human Rights*	467	7.2
2nd	*BMC International Health And Human Rights*	388	6.0
3rd	*The Lancet*	215	3.3
4th	*Medicine And Law*	89	1.4
5th	*International Journal Of Human Rights In Healthcare*	78	1.2
6th	*Reproductive Health Matters*	68	1.0
7th	*American Journal Of Public Health*	60	0.9
8th	*Journal Of Law Medicine And Ethics*	53	0.8
9th	*European Journal Of Health Law*	49	0.8
10th	*Social Science And Medicine*	48	0.7
	Total	1515	23.3

### Geographical distribution and active countries

**Fig. 3:**
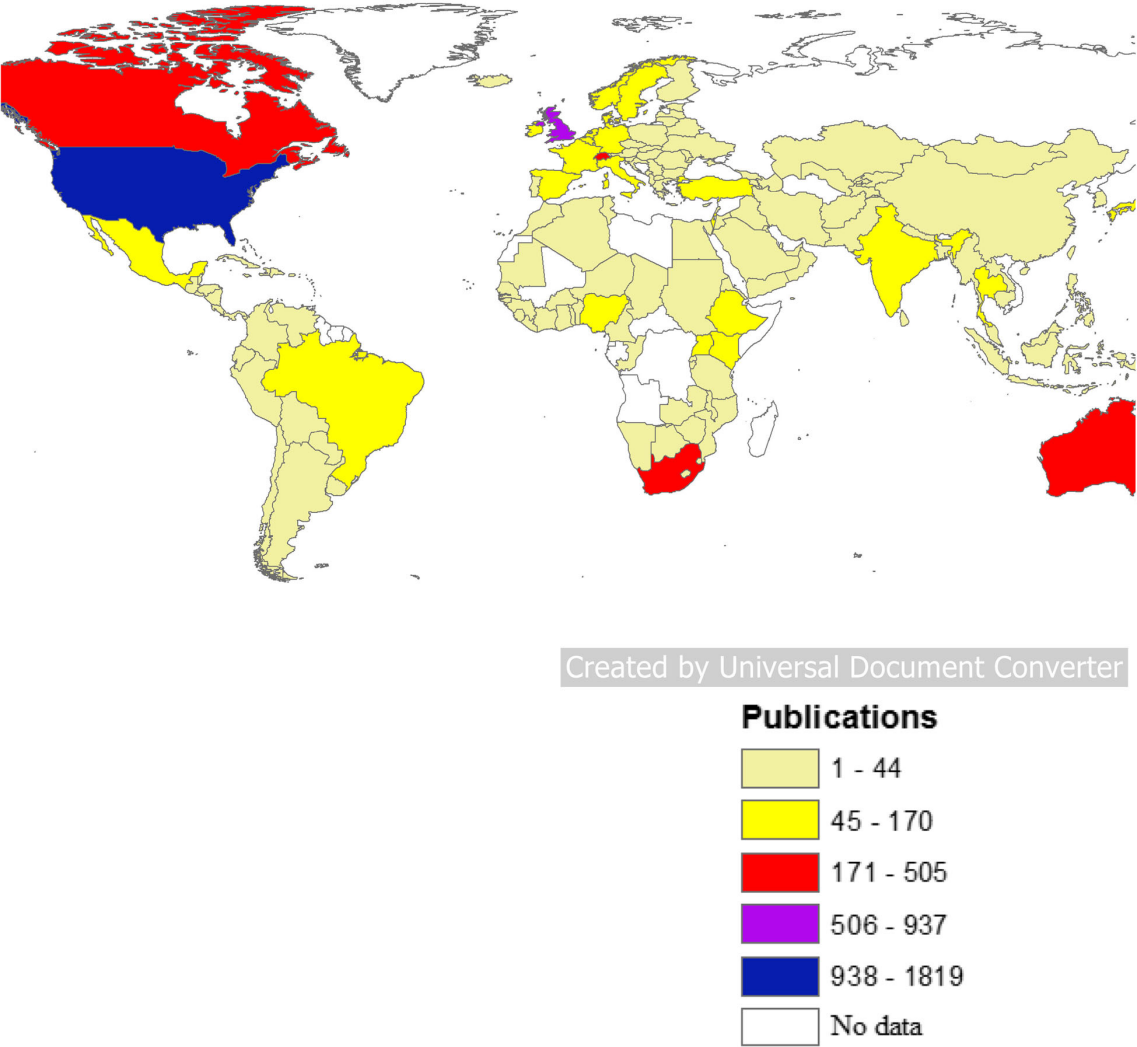
Geographical distribution of publications on HHR (1900–2017). The color coding is as follows:

Geographical distribution of retrieved documents was shown in Fig. 3. The map shows that most world countries participated in publishing the retrieved documents. Several countries played a key role in publishing on HHR. The USA led with the highest proportion of publications (*n* = 1819; 27.9%) followed distantly by the United Kingdom (UK) (938; 14.4%). The top 10 active countries included ones from different world regions such as North America, Africa, South America, Asia, and Western pacific region (Table 2). The top 10 active list included seven high-income countries and three upper-middle income countries (India, Brazil, and South Africa). The top 10 active countries contributed to 4972 (76.3%) documents. However, 2196 (44.2%) documents were published by inter-country collaboration, which represented an overlap in research productivity among collaborating countries.

**Table 2:** Top 10 active countries in publishing documents on HHR

Country	Number of publications	% *N* = 6513	Number of publications with intra-country collaboration (%)	Number of publications with inter-country collaboration (%)
United States	1819	27.9	1143 (62.8)	676 (37.2)
United Kingdom	938	14.4	542 (57.8)	396 (41.2)
Canada	505	7.8	244 (48.3)	260 (51.7)
Australia	456	7.0	284 (62.3)	172 (37.7)
South Africa	370	5.7	180 (48.6)	190 (51.4)
Switzerland	291	4.5	90 (30.9)	201 (69.1)
India	170	2.6	92 (54.1)	78 (45.9)
Brazil	152	2.3	93 (60.8)	59 (38.8)
Netherlands	142	2.2	69 (48.6)	73 (51.4)
Sweden	129	2.0	39 (30.2)	90 (69.8)
Total	4972	76.3	2776 (55.8%)	2196 (44.2%)

There is 44.2% overlap in publications across countries in the active list due to international collaboration

**Fig. 4:**
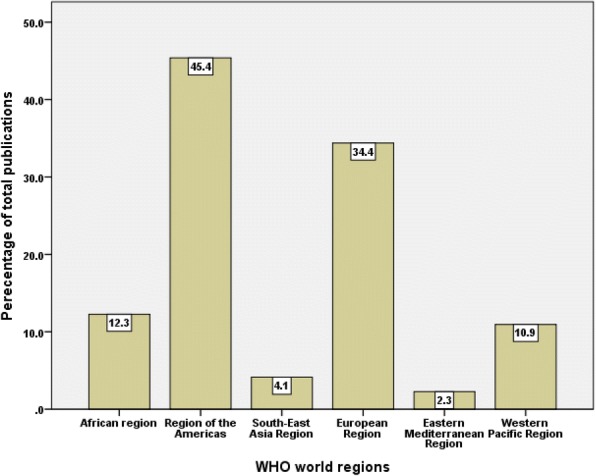
Distribution of publications on HHR based on WHO world regions (1900–2017)

**Fig. 5:**
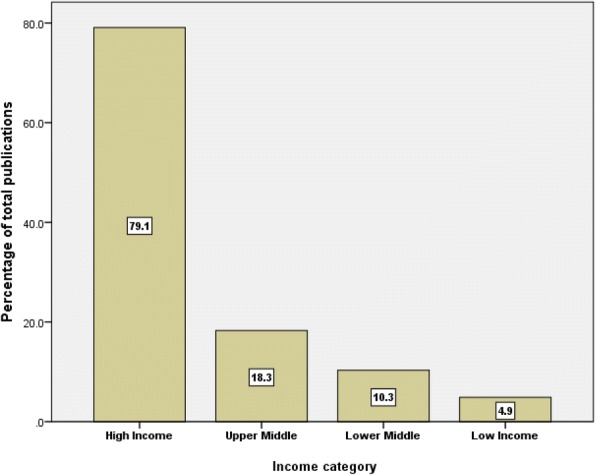
Distribution of publications on HHR based on World Bank criteria for income (1900–2017)

When research output was examined based on WHO world region, the Americas had the highest share followed by Europe while the regions of the Eastern Mediterranean and South-East Asia had the least share (Fig. 4). When productivity was examined by income, high-income countries contributed to approximately 79.1% of the retrieved documents, upper-middle-income countries contributed to approximately 18.3%, lower-middle-income countries contributed to approximately 10.3% of the retrieved documents, while low-income countries contributed to approximately 4.9% of the retrieved documents (Fig. 5). There were approximately 12.5% overlap in publications between high-income and LMIC. In total, there were 1463 (22.5%) documents with senior author being from LMIC. When data were standardized by population size, the research output from high-income countries was approximately four documents per one million inhabitants. For middle-income countries, the research output was 0.3 document per one million inhabitants. For low-income countries, the research output was 0.5 document per one million inhabitants.

### The most active institutions

Harvard University had the highest research productivity (*n* = 235; 3.6%) followed by the University of Toronto (*n* = 209; 3.2%) and World Health Organization (*n* = 171; 2.6%). When the retrieved data were limited to documents produced by LMIC, academic institutions based in South Africa were most active in this field and included University of Cape Town (*n* = 58), University of Witwatersrand (*n* = 32), and University of KwaZulu-Natal (*n* = 25).

## Discussion

The focus of the current study was on quantitative analysis and research trends in the field of HHR. The current study indicated a noticeable increase in the number of publications on HHR in the past two decades. Topics discussed in this field were diverse, but HIV/AIDS was most prominent. The Mediterranean region, with most history of conflicts, had the least contribution. High-income countries were leading in this field. Retrieved documents were published in journals in the field of human rights, law, and public health. The findings of the current study are discussed below.

### Geographical distribution of publications

The geographical distribution of publications on HHR was nearly global but with variations in size of research output across the world. Furthermore, geographical distribution was extensively more spread than that obtained for publications in other fields such as poverty or mobile health or global emerging pathogens [15, 21, 30]. The current study indicated that despite the USA was leading in this field; other non-American, non-European countries such as Brazil, India, and South Africa made noticeable contributions. The Mediterranean region with a long history of conflicts, wars, human abuse, torture, lack of democracy, poverty, inequalities, and massive numbers of refugees, has the least contribution to the HHR field. Currently, the dramatic changes in the Mediterranean region and the evolving Arab Spring should encourage political and cultural reform accompanied by research activities that should shed lights on gaps pertaining to HHR in this region. Relatively low research productivity from certain world regions and countries in Africa, Middle East, and Southeast Asia could be attributed to the limited number of academics and researchers in the field of human rights in general or could be due to lack of financial resources needed to support publication [31, 32].

The majority of retrieved publications were accomplished by high-income countries in the Americas and Europe. This was unsurprising given that research in these countries has made great progress in the past several decades. Furthermore, the academic regulations regarding tenure in academic institutions in the USA and other developed countries require research efforts to be carried out by researchers and academicians. Such academic regulations of tenure might be absent or loosely followed in many developing countries.

### Citation analysis

The fact that the *h*-index of retrieved articles was 88 indicates large number of citations and large number of readers. For example, the *h*-index for publication on health-related literature in refugees and asylum seekers was reported to be 64 [33] and that for female genital mutilation was 37 [34]. Another potential explanation for the relatively high *h*-index is the possibility of extensive self-citations due to the relatively small number of publications and researchers in this field compared to other fields.

### Growth of publications

Our results indicated that the past two decades had witnessed a dramatic increase in number of publications which could be attributed to increase in human rights violations due to wars, conflicts, torture, human trafficking, refugees, discrimination, increased prevalence of HIV in Africa, violence against women, stigma toward mental health disorders, and others [35–38]. The first and most clear overlap of human rights and health was observed in 1980s with the rise of HIV/AIDS and the appearance of discrimination and injustice suffered by people affected with HIV/AIDS [39–42]. This might partly explain the high number of retrieved documents in human rights and HIV/AIDS shown in our study. The presence of South Africa in the top active list of countries in human rights and health research could be attributed, in part, to the political and social movements that endorsed health rights among HIV people as a human right principle [43–53]. In South Africa, the political and economic reform made by late Nelson Mandela had a positive impact on research output in human rights in general and on HHR in particular [54, 55].

### Most active journals

The top active journals in publishing research in HHR included journals between the discipline of health and human right. The retrieved documents included editorials, which might affect the ranking of journals in the field of HHR. Exclusion of editorials from the bibliometric analysis could change the ranking of most active journals in the field of HHR. The top two human rights journals were launched in the past 16 years, which is another reason for the rise in the number of publications seen after the year 2000. The involvement of public health journals such as *The Lancet* and *American Journal of Public Health* in human rights emphasized the overlap between public health and human rights discipline. Journals in the field of law and medicine/health were also among top ten active journals. The legal aspects of human rights violations of health and access to treatment and medicines were mostly brought up by journals specialized in law and medicine/health [56–63].

### Most active institutions

The Harvard University ranked first as an institution and this could be attributed to the intensive involvement of researchers in Harvard University in this field which has led to launching a specialized journal in human rights (*Health and Human Rights*) published by Harvard School for Public Health. It must be noted here that it is the activity, commitment, and enthusiasm of both individual researchers and the administrative policies of the institution, which drive the high ranking of Harvard University. Furthermore, the presence of specific courses on HHR in the curriculum and teaching HHR concepts in classrooms are the major driving force for research output on HHR and the birth of new young researchers in HHR field.

### Bibliometric analysis versus systematic reviews

Bibliometrics is a field of increasing interest and popularity. Bibliometrics need to be carried out in all fields pertaining to health to encourage researchers and academics to get more involved in topics that need more attention and which can advance the health standards of minorities and disadvantaged people. In bibliometric analysis, the investigated research question is the volume of published research, how this volume of literature evolved with time, what major topics were of high interest, and the scientific impact of literature in a particular subject. However, in systematic reviews, a complete and exhaustive summary of current literature obtained from several electronic databases and relevant to a research question is provided. In systematic reviews, there are pre-determined criteria for inclusion of documents since the ultimate summary will be based on a relatively small number of articles that fit the predetermined criteria, which relate back to the main research question. Systematic reviews might come up with new data when utilizing meta-analysis. However, this is not the case with bibliometric analysis. New statistical data can be obtained from systematic reviews when meta-analysis is applied to the refined literature while no such meta-analysis technique is applied in bibliomeric analysis.

### Related studies

Systematic review, editorials and research articles that assessed the literature on HHR have been carried out, but none was carried out as a bibliometric analysis and mapping. A systematic review of literature on HHR concluded that the number of publications on HHR is increasing and new topics are being addressed [11]. Another study assessed the number of publications on HHR from Japan and concluded that literature in this field is increasing [13]. An editorial documented an increased attention of biomedical journals to human rights–related topics over time [12]. It should be emphasized here that bibliometric analysis in health inequalities or gender disparities have been published but none in HHR [64, 65]. Therefore, our study, to the best of author’s knowledge, is the first to do such analysis.

### Limitations

This study has few limitations, which need to be listed. First, no bibliometric study is 100% comprehensive since in bibliometric analysis one database was used to achieve the goal of the study. Second, given the large number of retrieved documents, no manual check could be carried out and the potential for false positive and false negative remains a possibility. However, the results obtained from interclass correlation suggest that our search strategy was reliable with less than 1% margin of error. This means that of the total retrieved documents, less than 60 could be false positive or false negative. Third, the search strategy used in this study was optimized to be highly reliable and valid, but potential minor mistakes in the search strategy cannot be ruled out completely. Finally, the most active authors and institutions need to be carefully interpreted due to the overlap of publications, research networking, self-citations, the mobility of researchers and academic staff from one institution to another. The ranking method used might have underestimated the productivity of some authors and instructions who do not work within research groups or networks. Usually, research networks and co-authorships apparently increase the productivity of certain institutions and authors, which should be taken into consideration when reading through the results presented in this study in which several authors exist within the same institution or network. Finally, active researchers who have different name format or spelling will end up with scattered research output that might not be added up. Therefore, such authors might not show up in the active list.

## Conclusion and recommendations

Calls by international organizations and international agreements for HHR had made several success stories in fighting diseases such as malaria and AIDS in many vulnerable and marginalized nations [66–71]. However, there is still more to be done to guarantee the right to health for all and to achieve the SDGs. There are still many disadvantaged nations or marginalized groups of people, globally and within countries, who do not enjoy rights to health. There are hundreds of millions of refugees in the Middle East [72, 73], children in Africa [74, 75], abused women all over the world [76, 77], and HIV affected people [78–80] who are still deprived of the basic human right to healthcare and health services. Researchers have an important role to play in achieving HHR by pointing to important neglected health topic or health needs of marginalized people, globally and within countries. Furthermore, researchers in Low and Middle-income countries have an important role in advocating health for all by encouraging politicians to adopt the concepts of human rights and right to health as part of national political agenda.

## Additional file

Additional file 1: Research strategy and keywords used to retrieve documents on HHR. (DOCX 17 kb)

## Abbreviations

HHR: Health and human rights
SDG: Sustainable Development Goals
WHO: World Health Organization
