# Supplementary material for: A bibliometric analysis of global research output on health and human rights (1900–2017)
Source: Glob Health Res Policy. 2018 Oct 22;3:30. doi: 10.1186/s41256-018-0085-8 (PMC6196451; doi:10.1186/s41256-018-0085-8)
Supplement: Supplementary file 1 — Research strategy and keywords used to retrieve documents on HHR. (DOCX 17 kb) [file 41256_2018_85_MOESM1_ESM.docx]

**Additional file 1**

|  | **Strategy** | **keywords** | **Number obtained** |
| --- | --- | --- | --- |
| **1** | Title search for the phrases: “health rights” or “right to health” | TITLE ("health right*" OR "right* to health") | 869 |
| **2** | Title search for the keywords (human rights AND health) | TITLE ("human right*" AND health*) | 1136 |
| **3** | Search for articles published in journals specialized in health rights and health | SRCTITLE ("human right*" AND health) | 989 |
| **4** | Search for articles published in journals specialized in human rights limited by the presence of the term health in title/abstract/key and by the affiliation of at least one author in a health – related department | (SRCTITLE ("human right*") AND TITLE-ABS-KEY (health) AND AFFIL (health OR medicine OR law OR psychology OR nursing OR "human right*")) | 759 |
| **5** | Title search for the phrase “human rights” limited by the presence of health-related terms in title/abstract/key | ( TITLE ( "human right*" ) AND TITLE-ABS-KEY ( "health* system*" OR torture OR "minority health" OR "health of minorit*" OR "child* health" OR "healthcare delivery" OR "access to medic*" OR "access to health*" OR "delivery of health*" OR "health* service*" OR "access to treatment" OR "reproducti* health" OR "Health disparit*" OR "health *equalit*" OR "health *equit*" OR "*justice in health" OR "health *justice" OR "violence against women" OR "women* health right*" OR "health status disparit*" OR health* OR "food *security" OR "people with disabilit*" OR "disabled person*" OR "disable* people" OR "health* discrimination" OR "genital mutilation" OR drug* OR "disease prevention" OR infect* OR disease OR "sexual right*" OR stigma OR "mental health" OR hiv OR "medical ethics" OR embryo OR cloning OR fetal ) ) | 3710 |
| **6** | Title search for the phrase “human rights” limited by the affiliation of one of the authors in a health – related department. | (TITLE ("human right*") AND AFFIL ("public health" OR "community health" OR "family medicine" OR health)) | 855 |
| **7** | Title-Abstract research for the phrase “human rights” limited by publication in a journal in the field of law and health | ((TITLE-ABS ("human right*") AND SRCTITLE (health AND law) )) | 320 |
| **8** | Title-Abstract research for the phrase “human rights” limited by publication in a journal in the field of health equity, health inequality, or medicine and law. | (((TITLE-ABS ("human right*") AND SRCTITLE (medicine AND law)))) OR (((TITLE-ABS ("human right*") AND SRCTITLE (health AND equity)))) OR (((TITLE-ABS ("human right*" ) AND SRCTITLE ( health AND *equalit* ) ))) | 309 |
| **9** | Title-abstract search for the phrase “human rights” limited by the presence of health-related terms in title/abstract/key and affiliation of one of the authors in a health – related department | ( TITLE-ABS ( "human right*" ) AND ( TITLE-ABS-KEY ( "health* system*" OR torture OR "minority health" OR "health of minorit*" OR "child* health" OR "health* delivery" OR "access to medic*" OR "access to health*" OR "delivery of health*" OR "health* service*" OR "access to treatment" OR "reproducti* health" OR "Health disparit*" OR "health *equalit*" OR "health *equit*" OR "*justice in health" OR "health *justice" OR "violence against women" OR "partner violence" OR "women* health right*" OR "health status disparit*" OR health* OR "food *security" OR "people with disabilit*" OR "disabled person*" OR "disabled people" OR "health* discrimination" OR "genital mutilation" OR drug* OR "disease prevention" OR infect* OR disease OR "sexual right*" OR stigma OR "mental health" OR health OR hiv OR "medical ethics" OR embryo OR cloning OR fetal ) AND AFFIL ( health OR medicine OR law OR nursing OR psychology OR "human right*" OR clinical ) )) | 4211 |
| **10** | Combination of strategies from 1 to 9 | **8,027** | |
| **11** | Exclude false positive articles | AND NOT TITLE-ABS ( diplomatic OR ventric* OR coronary OR atria OR cardiac OR heart OR "right brain" OR nerve OR neural OR neuronal OR hemisphere OR aorta OR brain OR "medical profession" OR pedsql OR anatomy OR anatomical OR electro* OR "court" OR "medical research" OR "scientific research" ) AND NOT SRCTITLE ( "american journal of political science" OR "international studies quarterly" OR "american anthropologist*" OR politics OR political OR peace OR anthropology )  **= 6845** | |
| **12** | Exclude publications in 2018 | **6624** | |
| **13** | Limit source type to journal articles | **6513** | |
